# Supplementary figures and images for: Analysis and comparison of the bacterial σ54 regulon: Evidence of phylogenetic trends in gene regulation
Source: PLoS One. 2025 Aug 1;20(8):e0327805. doi: 10.1371/journal.pone.0327805 (PMC12316311; doi:10.1371/journal.pone.0327805)

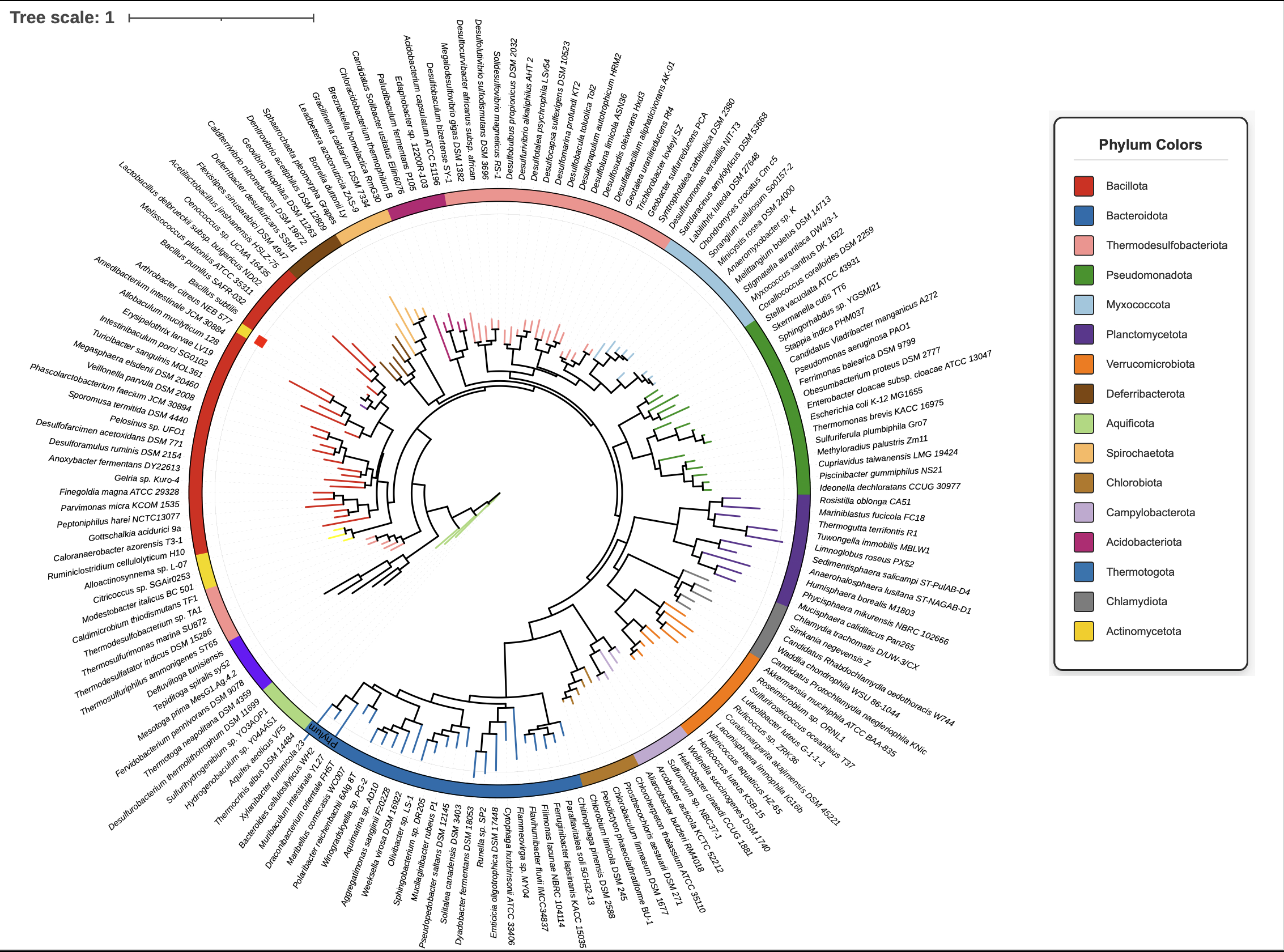

Supplement: S1 Fig — The 16S rRNA sequences from five representative organisms within each of the 33 phylogenetic classes included in our study were aligned and used to construct the phylogenetic tree shown in the figure. This tree reveals that Arthrobacter citreus NEB 577 clusters more closely with members of the Bacillota phylum (red) than with those of the Actinomycetota (yellow). (TIF) [file pone.0327805.s001.tif]

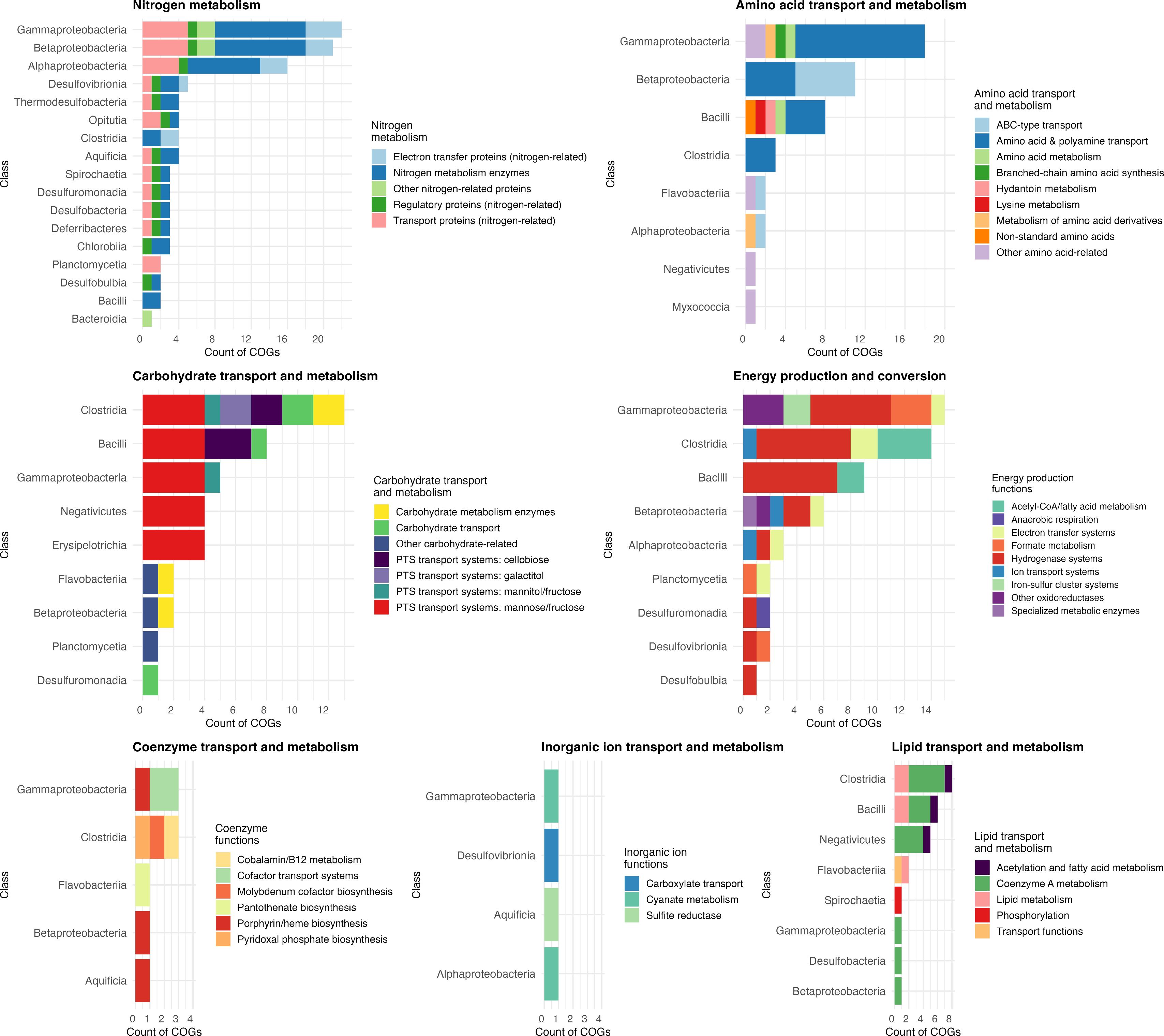

Supplement: S2 Fig — The bar chart illustrates the number of COGs per phylogenetic class, organized by their functional descriptions. Metabolism-related COGs are further subdivided into functional subcategories to highlighting enriched classes. The COGs included in each category were selected based on their statistical enrichment values, as determined by hypergeometric distribution statistics, as detailed in Fig 5. (TIF) [file pone.0327805.s002.tif]

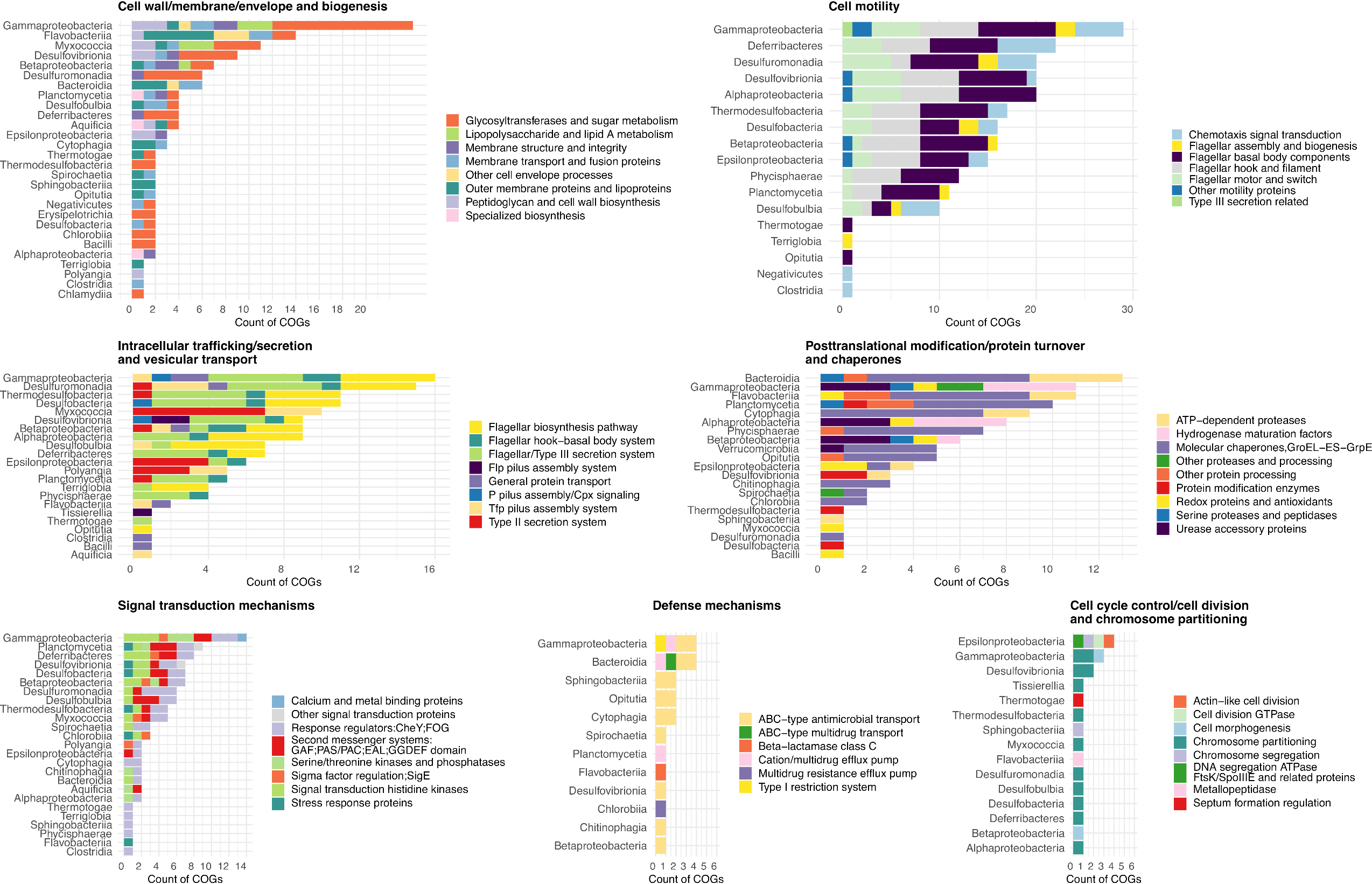

Supplement: S3 Fig — The bar chart illustrates the number of COGs per phylogenetic class, organized by their functional descriptions. Cellular processes and signaling-related COGs are further subdivided into functional subcategories to highlighting enriched classes. The COGs included in each category were selected based on their statistical enrichment values, as determined by hypergeometric distribution statistics, as detailed in Fig 6. (TIF) [file pone.0327805.s003.tif]
