# Supplementary material for: Analysis and comparison of the bacterial σ54 regulon: Evidence of phylogenetic trends in gene regulation
Source: PLoS One. 2025 Aug 1;20(8):e0327805. doi: 10.1371/journal.pone.0327805 (PMC12316311; doi:10.1371/journal.pone.0327805)
Supplement: Attachment — Submitted filename: Rebuttal_letter_sigma54_6_June.docx [file pone.0327805.s015.docx]

Dear Editor,

We sincerely thank you and the reviewers for your thoughtful and constructive comments. Your feedback was instrumental in improving the clarity, depth, and overall quality of our manuscript. We have carefully considered each suggestion and made the corresponding revisions, which we believe have significantly strengthened the work. We are grateful for the time and effort you devoted to the review process and for helping us enhance the impact of our study.

Journal requirements:

When submitting your revision, we need you to address these additional requirements.

1.  Please ensure that your manuscript meets PLOS ONE's style requirements, including those for file naming. The PLOS ONE style templates can be found at

[https://journals.plos.org/plosone/s/file?id=wjVg/PLOSOne_formatting_sample_main_body.pdf](https://track.editorialmanager.com/CL0/https:%2F%2Fjournals.plos.org%2Fplosone%2Fs%2Ffile%3Fid=wjVg%2FPLOSOne_formatting_sample_main_body.pdf/1/010f0195804bf65a-d3176826-6941-48be-a0a7-a0ecdb8d5459-000000/HtDk0nDj2U_A8ZwGX0W2MCJKMooC_fSNu8S75pJ5pqA=201)  and

[https://journals.plos.org/plosone/s/file?id=ba62/PLOSOne_formatting_sample_title_authors_affiliations.pdf](https://track.editorialmanager.com/CL0/https:%2F%2Fjournals.plos.org%2Fplosone%2Fs%2Ffile%3Fid=ba62%2FPLOSOne_formatting_sample_title_authors_affiliations.pdf/1/010f0195804bf65a-d3176826-6941-48be-a0a7-a0ecdb8d5459-000000/9OXYvqJnNopyaXXMevZAFkauOLGroAYjoWg-lo7YHaM=201)

Response: We have verified that the submission complies with PLOS ONE’s style requirements, including those related to file naming.2. Thank you for stating the following financial disclosure:

“Our work was supported by DGAPA-Universidad Nacional Autónoma de México (UNAM) PAPIIT (Grant IN222423); awarded to E.M. M.C. is a Ph.D. student enrolled in the Programa de Doctorado en Ciencias Bioquímicas-UNAM and recipient of a CONACYT fellowship (731966).”

Please state what role the funders took in the study.  If the funders had no role, please state: "The funders had no role in study design, data collection and analysis, decision to publish, or preparation of the manuscript."

If this statement is not correct you must amend it as needed.

Please include this amended Role of Funder statement in your cover letter; we will change the online submission form on your behalf.

Response: In the revised version of our cover letter, we have added the following statement: “The funders had no role in study design, data collection and analysis, decision to publish, or preparation of the manuscript.”

3. We note that you have included the phrase “data not shown” in your manuscript. Unfortunately, this does not meet our data sharing requirements. PLOS does not permit references to inaccessible data. We require that authors provide all relevant data within the paper, Supporting Information files, or in an acceptable, public repository. Please add a citation to support this phrase or upload the data that corresponds with these findings to a stable repository (such as Figshare or Dryad) and provide and URLs, DOIs, or accession numbers that may be used to access these data. Or, if the data are not a core part of the research being presented in your study, we ask that you remove the phrase that refers to these data.

Response: The phrase “data not shown” appeared only once in the previous version of our manuscript. As recommended, we have removed it, since the referenced data were not essential to the main findings of the article (see line 336 of our track-changes document).

[Note: HTML markup is below. Please do not edit.]

Reviewers' comments:

Reviewer's Responses to Questions

**Comments to the Author**

1. Is the manuscript technically sound, and do the data support the conclusions?

The manuscript must describe a technically sound piece of scientific research with data that supports the conclusions. Experiments must have been conducted rigorously, with appropriate controls, replication, and sample sizes. The conclusions must be drawn appropriately based on the data presented.

Reviewer #1: Yes

Reviewer #2: Yes

2. Has the statistical analysis been performed appropriately and rigorously?

Reviewer #1: No

Reviewer #2: Yes

3. Have the authors made all data underlying the findings in their manuscript fully available?

The [PLOS Data policy](http://track.editorialmanager.com/CL0/http:%2F%2Fwww.plosone.org%2Fstatic%2Fpolicies.action%23sharing/1/010f0195804bf65a-d3176826-6941-48be-a0a7-a0ecdb8d5459-000000/mFhq25xKH502cx3AIADtj9YMkyLIzF4RZUfezNKVHT8=201) requires authors to make all data underlying the findings described in their manuscript fully available without restriction, with rare exception (please refer to the Data Availability Statement in the manuscript PDF file). The data should be provided as part of the manuscript or its supporting information, or deposited to a public repository. For example, in addition to summary statistics, the data points behind means, medians and variance measures should be available. If there are restrictions on publicly sharing data—e.g. participant privacy or use of data from a third party—those must be specified.

Reviewer #1: Yes

Reviewer #2: Yes

4. Is the manuscript presented in an intelligible fashion and written in standard English?

PLOS ONE does not copyedit accepted manuscripts, so the language in submitted articles must be clear, correct, and unambiguous. Any typographical or grammatical errors should be corrected at revision, so please note any specific errors here.

Reviewer #1: Yes

Reviewer #2: Yes

5. Review Comments to the Author

Please use the space provided to explain your answers to the questions above. You may also include additional comments for the author, including concerns about dual publication, research ethics, or publication ethics. (Please upload your review as an attachment if it exceeds 20,000 characters)

Reviewer #1: The authors present a study titled “Analysis and comparison of the bacterial σ54 regulon: evidence of phylogenetic trends in gene regulation.” This work provides a comprehensive analysis of the presence of Sigma54 binding sites across a diverse range of bacterial species. The results are original, as the authors effectively demonstrate the distribution of these regulons across phylogenetic classes using their own regulon identification method. This approach offers valuable insights into evolutionary trends in gene regulation.


1. Comprehensive and review style introduction to Sigma54.

Response: We have expanded the Introduction section of our manuscript to provide a more comprehensive and review-style overview of σ⁵⁴. Additionally, we have carefully revised its structure and writing style to enhance clarity and coherence.

2. l.200 The R script used to calculate COG overrepresentation is not provided. Please include it to ensure reproducibility.

Response: To evaluate the statistical significance of the observed enrichment of genes regulated by σ⁵⁴ in specific COG categories across different phylogenetic clades, we used a custom PERL script that utilizes the *phyper* and *dhyper* functions from the R package. This script is publicly available via a Figshare repository, as referenced in the **Methods** section (lines 221 and 222 of our track-changes document).

3. l.207 Identification of EBPs is based on the presence of the PF00158 domain. However, it is unclear how specific this domain is. Could there be potential false positives? Provide an error analysis supporting the specificity of this domain

In response to the referee’s concern regarding the specificity of our EBP identification results, we conducted a more detailed inspection of the sequences initially identified as true positives in our *hmmsearch* analysis using the Pfam domain PF00158 (Sigma54_activat). We found that, despite strong sequence conservation and statistically significant E-values, some sequences lacked the “GAFTGA” motif, which is essential for interaction with the σ⁵⁴-RNA polymerase complex (39, 40).

Based on this observation, we adopted a more stringent approach to eliminate false positives from our EBP dataset. This revised pipeline integrates both, domain-based identification (via PF00158) and domain architecture analysis, as described in the **Identification of σ54-specific EBPs** of the **Methods** section**.** The steps are as follows:

1. **Initial EBP Identification:** we searched for the Pfam domain PF00158 (Sigma54_activat) within the Pfam database. This domain is known to be conserved in EBP proteins, which are involved in ATP-dependent interactions with σ54. In our search, we employed an E-value threshold of 1e-51 to ensure the inclusion of EBP candidates with high confidence.

**2. Sequence Redundancy Reduction:** The sequences containing PF00158 identified in the previous step were clustered using CD-HIT program (62).

**3. Motif Discovery:** The resulting non-redundant sequences were analyzed using MEME to identify conserved motifs.

**4.** **Motif Scanning Across Proteomes:** We then used MAST to scan for the presence of the 10 motifs discovered by MEME in the full set of proteomes.

**5. Filtering of sequences for the presence of the GAFTGA motif**. Considering that the “GAFTGA” motif have been found in functionally EBP proteins, we select all sequences containing this essential motif.

**6. Final Filtering of High-Confidence σ⁵⁴-specific EBPs:** Finally, we parsed the MAST output to retain only those sequences that contained all 10 motifs identified in the step #3 arranged in the following conserved order:

(2) – (10) – (6) – (3) – (4) – (9) – (7) – (5) – (8) – (1)

Sequences that meet all the previous filters were considered as bona-fide σ⁵⁴-specific EBP proteins.

As a negative control for our protocol to identify σ⁵⁴-specific EBP proteins, we analyzed the proteomes of 380 Archaea and 36 Eukaryota (Fungi), comprising a total of 1,169,896 proteins. Our results demonstrate 100% specificity, as no σ⁵⁴-specific EBPs were detected within this negative control set.

Based on our new results, we have updated the **Identification of EBPs** (lines 229–255) and the **Phylogenetic Distribution of EBPs** (lines 291 to 307) sections in the revised version of our article. The list of organisms used as a negative control is provided in Supplementary Table S4.

4. l.233 Please add the webpage link here: <https://biocomputo.ibt.unam.mx/arpondb/>. The web server is up and running, and the data is easily accessible.

We have confirmed that the web server is functioning as expected. Additionally, we have included the link to our webpage at the end of the **Abstract** section (lines 19 and 20 of our track-changes document).

5. l.240 Phylogenetic distribution of σ54: The phylogenetic group Actinomycetota appears to have only four organisms with a Sigma54. Could these 4 be false positives? How do these four organisms differ? Additionally, how confident are you that the AID, CBD, and DBD domains are not misidentified (are false positives)?

In response to the referee’s concern regarding the authenticity of the four σ⁵⁴-encoding organisms, we performed a multiple sequence alignment of their σ⁵⁴ proteins alongside those from four well-characterized reference species. To quantitatively assess sequence conservation, we calculated pairwise similarities between the AID, CBD, and DBD domains of the Actinomycetota sequences and the reference σ⁵⁴ proteins. The resulting similarity values are compiled into square matrices and presented in a new supplementary table (S6 Table). In addition, comparable matrices based on Pfam domain bitscores are provided in S7 Table.

A careful examination of the data revealed that, among the original set of four Actinomycetota organisms with putative RpoN proteins, only *Arthrobacter citreus* NEB 577 encodes an RpoN protein with significant Pfam bit scores across all three conserved domains (AID, CBD, and DBD). Further analyses, including the construction of a 16S rRNA-based phylogenetic tree and a detailed review of NCBI taxonomic annotations, suggested a likely misclassification: *Arthrobacter citreus* NEB 577, originally assigned to Actinomycetota, appears to be more closely related with the class Bacilli.

Taken together, our findings support the conclusion that Actinomycetota, as currently classified, lacks a functional σ⁵⁴ sigma factor. Accordingly, we have revised the **Phylogenetic distribution of σ54** of the **Results and Discussion** section (lines 316 to 327 of our track-changes document).

6. l.286 Identifying protein function based on a single domain is problematic. While the approach is generally valid, it is likely to result in a significant number of false positives and false negatives. How specific is the Pfam Sigma54_activat motif (PF00158)? The results indicate up to 100 EBPs per genome—how realistic is this estimate? See also point 3

As explained in our response to comment 3, which raised a similar concern about the limited confidence in identifying σ⁵⁴ proteins based solely on a single Pfam domain, we have refined our search strategy. The revised analysis now employs a more rigorous approach that incorporates ten distinct motifs identified using the MEME program, filters sequences for the presence of the GAFTGA motif, and performs a comprehensive search across the proteomes of our non-redundant organism set. This updated procedure is detailed in the **Identification of σ⁵⁴-specific EBPs** (lines 229–255) of the **Methods** section, and **Phylogenetic Distribution of σ⁵⁴-specific EBPs** (lines 316–327) of the **Results and Discussion** section, of the revised manuscript.

7. l.321 The Matthew correlation coefficient (MCC) of 0.778 appears to be based on E. coli. Given the reported precision and specificity of 100%, this suggests an overfitted model for E. coli. How can such a model be reliably applied to genomes that are not closely related to E. coli? Error analysis is not provided

In response to the referee’s comment, we adopted a revised strategy to minimize potential bias toward *E. coli* or other model organisms in the construction of the initial seed matrix used at the onset of our iterative promoter search procedure. Specifically, we curated a diverse dataset of RpoN-dependent promoter sequences from the most frequently cited scientific publications and major publicly available regulatory databases. This dataset comprises 720 experimentally validated RpoN promoter sequences from 56 different organisms obtained from major regulatory databases, including RegPrecise [56], DBTBS [57], EcoCyc [58], and from the compilation of σ⁵⁴-dependent promoter sequences reported in Barrios et al. (1999) [59].

The initial set of RpoN promoter sequences was used to construct a seed position-specific scoring matrix (PSSM) using MEME. We then implemented an iterative motif discovery and refinement process, combining MEME and MAST. The seed PSSM was used in the initial MAST search, and in subsequent rounds, it was updated with matrices derived from taxon-specific sequences identified at each step. As noted previously, this cyclic procedure was applied independently to each of the 33 phylogenetic classes to enhance precision. We believe that this design, grounded in promoter data from phylogenetically diverse organisms, substantially reduces the risk of overfitting.

To compare the performance of our original protocol with the updated version, we used the same set of experimentally verified σ⁵⁴ promoters reported in EcoCyc. Both approaches yielded similar results for the Matthews correlation coefficient (0.778 vs 0.778), specificity (100% vs 100%), and sensitivity (60% vs 60%). As in our previous analysis, we attribute the high specificity of the predictions to the stringent significance thresholds applied, regardless of how the seed matrix was constructed. We believe that this high specificity ensured the retention of only high-confidence σ⁵⁴ promoter sequences as true positives.

In the **Methods** section (lines 173–190 of our track-changes document), we updated the description of the procedure used to generate the seed PSSM and made minor modifications to Fig 2 to reflect the improvements introduced at this initial step. Additionally, we now include the curated list of RpoN-dependent promoter sequences used in our analysis as a new supplementary table (Table S2), as well as the seed position-specific scoring matrices (PSSMs) used for the initial MAST searches in each of the taxonomic groups analyzed (Table S3). Furthermore, we revised lines 377–395 of the track-changes document to report the performance of our computational protocol using a new seed matrix generated from experimentally validated σ⁵⁴ promoter sequences in *Escherichia coli* and *Bacillus subtilis*, representing the Pseudomonadota and Bacillota phyla, respectively.

8. l.332 The values of the four parameters for the hypergeometric distribution depend heavily on the number of false positives and false negatives detected. If genomes from different taxonomic groups are included, the DNA and protein motifs will likely introduce more errors, which will impact the hypergeometric results. How confident are you that Sigma54 is being analyzed specifically, rather than general sequence diversity? Would a similar analysis with Sigma70 yield comparable trends? Was this tested?

As explained in our response to comment #7, the updated version of our procedure is based on the construction of an initial, curated set of experimentally validated σ⁵⁴-dependent promoter sequences from 720 experimentally validated σ54-dependent promoter sequences derived from 56 bacterial species. This selection was intentionally designed to avoid bias toward specific model organisms, such as *E. coli*. This set of seed sequences were used to build an initial seed MEME matrix.

With this seed MEME matrix, we started subsequent rounds of motif discovery and scanning were performed using updated position-specific scoring matrices derived from taxon-specific sequences identified in each iteration. As previously mentioned, this cyclic procedure was applied independently to each of the 33 phylogenetic classes to improve precision and reduce overfitting.

Using this computational protocol, we successfully identified, with statistically significant support, all known σ⁵⁴-regulated processes in the correct phylogenetic groups in which they were originally reported. We interpret these results as internal positive controls that support the reliability of the newly identified σ⁵⁴-regulated processes across specific phylogenetic clades.

Furthermore, we believe that non-specific or erroneous identification of σ⁵⁴ promoters would not result in the statistically significant enrichment of functionally related genes within the same metabolic pathway. Instead, such errors would likely yield scattered and unrelated hits across various functions. For this reason, we are confident that the statistically significant enrichments observed using the hypergeometric test are valid and reflect the true evolutionary distribution of σ⁵⁴-regulated metabolic processes.

Given these positive results and considering that σ⁷⁰ promoter sequences tend to be less conserved, often due to regulation by transcriptional activators (e.g., AraC, SoxS, MarA, PhoB) or repressors that overlap the promoter region, we did not include σ⁷⁰ promoter analysis as an additional control.

9. l.1.4 – l.1.8 The list of pathways is extensive. While some have literature references to support their regulation by Sigma54, in many cases, it is unclear whether these pathways are directly regulated. Providing additional context or evidence would improve clarity.

In light of the reviewer’s observation and with the aim of providing a clear and comprehensive source supporting the direct regulation of the pathways discussed in our study, we have made available through our web server aRpoNDB (<https://biocomputo.ibt.unam.mx/arpondb/>) and in the figshare repository (https://figshare.com/projects/Analysis_and_comparison_of_the_bacterial_54_regulon/251936) a downable zip file with the complete set of σ⁵⁴-dependent promoter sequences identified in our study. This file contains the following information:

a) All the σ⁵⁴-dependent promoter sequences identified in our analysis.
b) The corresponding σ⁵⁴-regulated operons, including for each gene its identifier, the COG annotation of its protein product classified by their cellular or biological process, and a functional description; and
c) The source of each promoter sequence, whether derived from the literature, publicly available databases, or identified in this study.

We believe that this supplementary file will serve as a valuable resource for supporting the conclusions of our work and for future research on σ⁵⁴-mediated transcriptional regulation.

10. l.477 The same issue applies to Cellular processes and signaling.

The concern raised here is addressed by the same supplementary resource described in our response to Comment 9. Specifically, Supplementary Table S10 includes σ⁵⁴-regulated genes associated with **Cellular processes and signaling**, along with their corresponding promoter sequences, functional classifications, and supporting references. We respectfully refer the reviewer to our response to Comment 9 for further details.

11. l.683 Similar concerns apply to Information storage and processing.

The concern raised here is addressed by the same supplementary resource described in our response to Comment 9. Specifically, Supplementary Table S10 includes σ⁵⁴-regulated genes associated with **Information storage and processing**, along with their corresponding promoter sequences, functional classifications, and supporting references. We respectfully refer the reviewer to our response to Comment 9 for further details.

12. Several web servers and computational tools are already available for mining Sigma54 regulons. However, the authors do not provide a comparison to existing tools or demonstrate whether their method offers any improvements. A comparative analysis would strengthen the study.

Motivated by the referee’s comment, we searched for the most recent web servers designed to predict σ⁵⁴ promoters to compare their performance with that of our computational protocol.

The first server evaluated was the ProPr54 web server (http://propr54.molgenrug.nl), published in March 2025 (doi:10.1093/nargab/lqae188). Unfortunately, despite repeated attempts, we were unable to obtain any results. The platform consistently failed to respond, even to basic operations such as downloading the examples .fna or .gff files.

The second server tested was iProm-Sigma54 (https://nsclbio.jbnu.ac.kr/tools/iProm-Sigma54), published in March 2023 (doi.org/10.3390/cells12060829). While the platform allowed us to analyze a small number of sequences via a direct input window, it consistently returned an “Internal Server Error” when attempting to upload larger sequence sets using the provided file upload option.

Given the limitations encountered with the ProPr54 and iProm-Sigma54 web servers, we opted to evaluate the performance of our method by comparing its Matthews correlation coefficient (MCC) with values reported for other computational approaches. We acknowledge that a rigorous benchmarking exercise would require the use of the same reference sequences for positive and negative controls across all methods. Nevertheless, we believe that an indirect comparison based on reported performance metrics can still provide meaningful context.

As indicated in the updated version of our article (lines 382-386), we assessed the accuracy of our predictions using experimentally validated σ⁵⁴ promoters from two phylogenetically distant model organisms, *E. coli* and *B. subtilis*, drawing on curated data from the EcoCyc and DBTBS databases. Our method achieved MCC values of 0.672 and 0.738, respectively. These values are within the performance range of other bacterial promoter prediction tools. For example, Shahmuradov et al. (2016, doi: 10.1093/bioinformatics/btw629) and Amin et al. (2020, doi: 10.1093/bioinformatics/btaa609) reported MCC values between 0.45 and 0.86 for ten different methods, with a mean of 0.63, a median of 0.61, and a mode of 0.61.

We would like to emphasize that, although the predictive performance of our method is comparable to existing tools, most previous studies have focused on identifying σ⁵⁴ promoters in a single organism or within narrow phylogenetic scopes. To the best of our knowledge, our study is the first to conduct a comparative analysis of σ⁵⁴ sigmulons across major bacterial phylogenetic clades and to assess their functional enrichment in metabolic and biological processes.

13. A clear overview is missing that directly compares known regulon-associated genes/proteins with those identified in this study. Additionally, there is no clear indication of genes previously unknown to be influenced by Sigma54. Such a summary would enhance the impact of the findings.

Motivated by the reviewer’s feedback, we compiled all σ⁵⁴-dependent promoters predicted in our study into a set of text files, organized hierarchically by major COG functional categories, associated cellular processes or metabolic pathways, and the COG assignments of the corresponding regulated genes. Each promoter entry includes its original source, whether from the literature, public databases, or newly identified in this study, as well as the predicted regulated operon. We hope this detailed dataset will serve as a valuable resource for future research into σ⁵⁴-mediated regulation. These files have been compressed into a ZIP archive which is available through our web server aRpoNDB (<https://biocomputo.ibt.unam.mx/arpondb/>)

To complement this dataset and provide a consolidated overview of our key findings, we have added a new section to the article (lines 915 to 924) summarizing the main results of the study. This summary is visually represented in the newly included Figure 9, which highlights the breadth and functional relevance of the predicted σ⁵⁴-dependent regulatory network across diverse phylogenetic groups.

14. l.834 "Through rigorous statistical analysis": The cross-organism application of PSSMs for motif identification in DNA is highly unreliable. The manuscript does not explain how errors were quantified or how confident the authors are in their results. Please provide details on this analysis.

We agree that the cross-organism application of PSSMs for DNA motif identification can be unreliable in some cases. However, we believe the reliability strongly depends on three key factors: a) the phylogenetic proximity of the organisms included in the analysis, b) the degree of sequence conservation in the element being identified (σ⁵⁴ promoters in our case), and c) the number of sequences used to construct the PSSMs.

These factors were carefully considered in our study. As explained in our previous response to question # 7 and detailed in the **Methods** section and illustrated in Fig 2, the initial seed matrix was built using a set of 720 experimentally validated RpoN-dependent promoter sequences from 56 different organisms. We then applied a cyclic pattern-finding and motif-discovery pipeline independently to each of the 33 phylogenetic classes included in our analysis. This approach was designed to improve both sensitivity and specificity, while minimizing bias toward any single model organism.

As described in the “**In silico identification of σ54-dependent promoters**” section (lines 377 to 410), the accuracy of our predictions was evaluated with a set of experimentally validated σ⁵⁴ promoters reported in the EcoCyc database and DBTBS. The performance of our method evaluating the EcoCyc database was as follows: Matthew correlation coefficient: 0.672, precision: 100%, specificity: 100% and sensitivity: 45%. The performance of our method evaluating the DBTBS was as follows: Matthew correlation coefficient: 0.738, precision: 100%, specificity: 100% and sensitivity: 99.8%.

The low sensitivity of our method can be attributed to two main factors. First, we applied stringent cutoff values to maximize specificity, thereby increasing the likelihood that all predicted σ⁵⁴-dependent promoters are true positives. Second, the method has limited capacity to detect promoters that diverge significantly from the consensus sequence. Supplementary Table S10 lists all experimentally validated σ⁵⁴ promoters that were not detected by our approach, along with a description of their specific deviations from the consensus.

In addition, as explained in our responses to Comments #7 and #8, our procedure successfully identified all known σ⁵⁴-regulated processes with statistically significant values and within the correct phylogenetic groups. We consider these results to serve as positive controls, supporting the reliability of the newly identified σ⁵⁴-regulated processes observed in specific phylogenetic clades, as well as the statistically significant enrichment of functionally related genes within the same metabolic pathways or cellular processes. In our view, inaccurate identification of σ⁵⁴ promoters would likely result in scattered, unrelated hits across different pathways. Therefore, we are confident that our matches represent genuine σ⁵⁴-regulated genes.

15. l.840 "Our study revealed unreported cellular processes": Could these findings be due to false positives? Additionally, expected genes are not explicitly discussed—were they identified as expected, or were some missed? These results should be included to clarify the reliability of the method.

As detailed in the **section In silico identification of σ54-dependent promoters** (lines 378-395), our method demonstrated to achieved 100% of specificity when tested again experimentally verified σ⁵⁴ promoters of *Escherichia coli* (EcoCyc) and *Bacillus subtilis* (DBTBS).

Reviewer #2: This is a very interesting survey of the sigma54 regulon across a very large set of genome sequences. It makes an important contribution to the field. The accompanying website is also a useful contribution.

However, I have some suggestions and comments that the authors may wish to consider.

The website at <https://biocomputo.ibt.unam.mx/arpondb/> is a good contribution. However, previous experience suggests that websites often "disappear" after publication as the authors move on to new projects, funding for webhosting dries up, etc. I strongly urge the authors to deposit a snapshot of the webpage in Zotero or as supplementary data attached to the manuscript.

We appreciate the referee’s suggestion. In response, and as indicated in our reply to comment #13 from Reviewer #1, we have compiled all σ⁵⁴-dependent promoters predicted in our study into a set of text files, organized hierarchically by major COG functional categories, associated cellular processes or metabolic pathways, and the COG assignments of the corresponding regulated genes. Each promoter entry includes its original source, whether derived from the literature, publicly available databases, or identified in this study, as well as the predicted regulated operon.

To facilitate accessibility and reproducibility, these files have been compressed into a ZIP archive and referenced in the subsection **In silico identification of σ⁵⁴-dependent promoters** within the **Results and Discussion** section of the revised manuscript. They are provided as supplementary material (Table S11). We hope this curated dataset will be a valuable resource for the community and support future research on σ⁵⁴-mediated transcriptional regulation across diverse bacterial taxa.

I am surprised that the authors do not cite this paper:

Barrios, H., Valderrama, B., & Morett, E. (1999). Compilation and analysis of sigma(54)-dependent promoter sequences. Nucleic acids research, 27(22), 4305–4313. <https://doi.org/10.1093/nar/27.22.4305>

This is a seminal paper that represents the first major attempt to systematically catalogue sigma54 promoter sequences and underpins much of the subsequent computer-based predictive studies of the sigma54 regulon.

We thank the referee for highlighting the pioneering work by Barrios et al. (1999) on the characterization of σ⁵⁴-dependent promoter sequences. This important study is now cited and discussed in the **Data preparation and construction of the seed matrix for σ⁵⁴-dependent promoters** of the **Methods** section (lines 173 to 190 of the revised manuscript).

As noted in our response to comment #7 from Reviewer #1, we incorporated the σ⁵⁴-dependent promoter sequences reported by Barrios et al. (1999), along with additional data from regulatory sequence databases such as RegPrecise, DBTBS, and EcoCyc, to generate the initial set of seed sequences. These sequences were used to construct the first MEME-derived position-specific scoring matrix (PSSM) employed in our iterative pattern discovery process using MEME and MAST.

The authors should also discuss this recent work and how it is similar/dissimilar the theirs:

Achterberg, T., & de Jong, A. (2025). ProPr54 web server: predicting σ54 promoters and regulon with a hybrid convolutional and recurrent deep neural network. NAR genomics and bioinformatics, 7(1), lqae188. <https://doi.org/10.1093/nargab/lqae188>

We thank the referee for recommending that we mention the recent work by Achterberg & de Jong (2025). (As stated in response to Reviewer #1 (comment #12), "We have included in the revised manuscript a new paragraph in the Results and Discussion section [lines 394-407] discussing other computational methods to identify σ⁵⁴-dependent promoter sequences, including the method mentioned above. However, although Achterberg and de Jong (2025) claim to have implemented the ProPr54 model as a ”broadly applicable tool for the prediction of σ⁵⁴ promoters and regulon genes in bacterial genome sequences”, we never got any results from the web server despite having tried it out several times. I could not get the server to respond for anything, even just downloading the examples of . fna or. gff files.

We encountered similar issues in utilizing iProm-Sigma54: a CNN server (https://nsclbio.jbnu.ac.kr/tools/iProm-Sigma54), released on March 2023 (doi. org/10.3390/cells12060829). The site did permit us to download few sequences with a direct input window; it constantly gave an “Internal Server Error” message when we tried to upload the larger sets of sequences using the file upload option provided.

Lines 117-125. When selecting genome sequences for their analyses, did they consider the quality/completeness/reliability of the genome sequences? If yes, then what quality criteria did they apply. If not, then why not?

As detailed in “Genome Sequence Datasets” subsection in the “Methods” section of our manuscript, the initial 6,889 genomes were found in the KEGG GENOME database, which only contained high-quality genomes highly complete and reliable. Thus, all selected genomes satisfy these conditions.

Lin 159. "propensity of some sigma factors for self-transcription". Is there any published evidence that sigma54 initiates transcription of itself? Given the uniqueness of this transcription factor, it is dangerous to extrapolate what we know about other sigma factors to sigma54. It would make more sense to start the seed matrix using a set of confirmed promoter sequences or a datasets such as that of Barrios et al. (1999).

Following the referee’s suggestion, and as noted in our response to Reviewer #1 (comment #7), the revised version of our manuscript includes an updated seed matrix generated from experimentally confirmed σ⁵⁴-dependent promoter sequences. This set includes the promoter sequence reported by Barrios et al. (1999), as well as sequences curated from the RegPrecise, DBTBS, and EcoCyc databases. As detailed in the **Data preparation and seed matrix construction** for σ⁵⁴-dependent promoters of Methods section’ the dataset used to create the starting seed matrix is composed by 720 σ⁵⁴-dependent promoter sequences, spread over 56 diverse organisms. This dataset is provided to readers as a new supplementary table (Table S2).

Line 247. Please be consistent in taxonomic names. Cereibacter sphaeroides is a synonym of Rhodobacter sphaeroides. So, use one name or the other. Don't use one synonym for one strain and another synonym for the other. Both names refer to precisely the same entity, so it makes no sense to use both. See <https://lpsn.dsmz.de/species/rhodobacter-sphaeroides> for full taxonomic information about this species.

We appreciate the referee’s valuable comment. In response to this observation, we have verified and corrected the official names of the organisms mentioned in the revised version of the manuscript. Additionally, since both strains belong to the same species, *Cereibacter sphaeroides*, we have retained only one to avoid redundancy. Accordingly, we have updated the first paragraph of the **Results and Discussion** section under **Phylogenetic distribution of σ⁵⁴** (lines 291 to 307 of our track-changes document) and revised Figure 3 to reflect this change.

Line 260. "Pfam motifs AID, DBD, and CBD". Are you sure that these are "motifs" in Pfam? Pfam has families, domains and clans. I don't think it has motifs. Also, please provide the Pfam accession numbers for these entities.

We appreciate the reviewer’s attention to this detail and have taken it into account. In the revised version of the manuscript, we have correctly referenced the relevant Pfam domains throughout the text, including PF00309 (AID), PF04552 (DBD), and PF04963 (CBD).

Line 273. "We hypothesize that this overlap in high GC content ...". To what extent has this been previously explored in the literature and to what extent does previous work support the hypothesis? For example, see:

Calistri, E., Livi, R., & Buiatti, M. (2011). Evolutionary trends of GC/AT distribution patterns in promoters. Molecular phylogenetics and evolution, 60(2), 228–235. https://doi.org/10.1016/j.ympev.2011.04.015

Although the low or absent frequency of the *rpoN* gene in Actinomycetota has been previously reported (9), to the best of our knowledge, no explanation has been proposed linking this absence to the high genomic GC content of these organisms and the similarly high GC content of σ⁵⁴ promoter consensus sequences The only association discussed with respect to the study cited is a negative correlation here σ⁵⁴ frequency genome size and see specifically mentioning that σ⁵⁴ is frequently absent in smaller genomes to include obligate endosymbionts or facultative intracellulars organisms. This comment is quoted in our revised article (lines 328–336 of our track-changes document).

On the other hand, although Calistri et al. (2011) explored evolutionary trends in GC/AT distribution within promoter regions, their conclusions, highlighting a prevalence of GC-rich promoters in eukaryotes and AT-rich promoters in bacteria, do not align with the high GC content observed in the promoter regions of Actinomycetota. For this reason, we chose not to include their study in our article.

Line 277. "Tree of Life [with capital initial letters]". What is this? Can the authors provide a reference or URL? Do they mean <https://www.evogeneao.com/en/explore/tree-of-life-explorer>? Or <https://itol.embl.de/>?

We appreciate the reviewer’s attention to this detail and have addressed it in the revised version of our article (line 334) by including a reference to the paper by Ivica Letunic and Peer Bork, ‘Interactive Tree of Life (iTOL) v6: recent updates to the phylogenetic tree display and annotation tool’ (2024). Reference (65).

Line 353. "Nitrogen metabolism has long been recognized as one of the key processes that is regulated by σ54, initially leading to the association of this sigma factor with the name σN. However, our study's findings challenge the notion that the regulation of nitrogen metabolism, which is indicated by the red circles in Fig 5, is a universal trend across bacteria.". Here, the authors are setting up a "straw man". I am not aware of any recent reviews that posit that sigma54's role in nitrogen regulation is universal. Can the authors cite any specific papers with which they disagree on this point?

We thank the reviewer for this thoughtful observation. We acknowledge that the original wording in our manuscript overstated the generality of σ⁵⁴’s role in nitrogen metabolism by suggesting it is a universal regulatory function across bacteria. While nitrogen metabolism is indeed one of the key processes regulated by σ⁵⁴ in many bacterial species, we recognize that this function is not conserved universally.

We agree with the reviewer that σ⁵⁴ has a broad and diverse range of regulatory roles beyond nitrogen metabolism, including control of motility, biofilm formation, stress responses, and other specialized functions depending on the organism. Accordingly, we have revised the text in lines 442 to 450 to more accurately reflect the variable and context-dependent nature of σ⁵⁴ function without suggesting a consensus that does not exist in the current literature.

Line 403. "Interestingly, as previously documented, RNAP-σ54 complex also regulates the transcription of PTS component genes in β-Proteobacteria. Also gamma-proteobacteria. See:

Studholme D. J. (2002). Enhancer-dependent transcription in Salmonella enterica Typhimurium: new members of the sigmaN regulon inferred from protein sequence homology and predicted promoter sites. Journal of molecular microbiology and biotechnology, 4(4), 367–374.

We thank the reviewer for this insightful observation. We have revised the **Carbohydrate transport and metabolism** section to clarify that the σ⁵⁴ regulates the transcription of PTS component genes in both β-Proteobacteria and γ-Proteobacteria. The suggested reference has been incorporated accordingly (line 495, reference 71, of our track-changes document). We also have included this reference in the **Cell wall/membrane and envelope biogenesis** section to comment the putative σ⁵⁴ regulation virulence-associated genes in members of the γ-Proteobacteria class (lines 603 to 604 of our track-changes document).

Line 507. When considering the sigma54 regulon in planctomycetes, the authors may wish to read this paper:

Studholme, D. J., & Dixon, R. (2004). In silico analysis of the sigma54-dependent enhancer-binding proteins in Pirellula species strain 1. FEMS microbiology letters, 230(2), 215–225. <https://doi.org/10.1016/S0378-1097(03)00897-8>.

We appreciate the reviewer’s suggestion and the valuable reference, which has helped us enhance our description of σ⁵⁴-dependent regulation in this phylum. As with the previous case, the corresponding reference has been incorporated in different parts of the revised version of our article (lines 396, 410, of our track-changes document)

I note that the authors' predictions at <https://biocomputo.ibt.unam.mx/arpondb/tables/rba_Rhodopirellula_baltica.html> are not entirely consistent with the results in that paper. The authors may or may not wish to consider that. Furthermore, I was unable to locate in the authors' web database their predictions for "Aquifex aeolicus" to compare those against the published work the sigma54 regulon in that organism.

Following the referee’s suggestion, we reviewed the consistency between our web page and the results presented in the manuscript.

Additionally, as a result of the improvements made to generate the seed MEME-derived position-specific scoring matrix (PSSM), as explained in our response to comment #7 from Reviewer #1 and in the section **Data preparation and construction of the seed matrix for σ⁵⁴-dependent promoters** (lines 173–190 of the revised manuscript), our updated program successfully identified nearly all predicted σ⁵⁴-dependent promoters of *Aquifex aeolicus* reported in doi:10.1128/JB.182.6.1616-1623.2000 and our predictions https://biocomputo.ibt.unam.mx/arpondb/tables/aae_Aquifex_aeolicus.html

Throughout the manuscript, please use a superscript for 54 when writing sigma54. And do the same for other sigma factors; e.g. sigma70 should have superscripted 70.

In response to this comment, we have revised the notation of σ⁵⁴ and other sigma factors to use superscript formatting.

Line 23 and elsewhere. Rather than saying "bacterial organisms", it is more straightforward to just say "bacteria".

We have replaced the term “bacterial organisms” with “bacteria” throughout the text of our article.

Line 25. Instead of "corresponds to" simply write "is".

The sentence on line 25 has been revised to improve its grammar and style.

Line 33. Delete "lacking sequence homology". First, it is unclear how sequence homology is different from simply "homology". Second, the lack of homology is discussed in subsequent sentences, so does not need to be stated here.

As suggested by the referee, the phrase “lacking sequence homology” has been removed from the text.

Line 44. "ubiquitously present". No. As the authors show later in this manuscript, this sigma factor is NOT ubiquitous. There are several bacterial taxa where this sigma factor is absent.

We thank the referee for their comment. The corresponding sentence has been revised to reflect the broad, but not ubiquitous, distribution of σ⁵⁴ across the bacterial kingdom.

Line 57. "class Clostridiales". This is an order, not a class.

We appreciate the reviewer’s correction and have revised the text to accurately refer to the class Clostridia.

Line 79. "matrices.". These are not matrices. They are profile-HMMs.

We have revised the corresponding sentence to accurately refer to Pfams as profile-HMMs.

Line 78. Do not use italics for Pfam.

We have removed the italic formatting from the word *Pfam* to follow proper style conventions.

Line 112. "1,249 organisms". Here, and many other times in the manuscript the authors refer to numbers of organisms. What does this mean? Does it mean numbers of species? Strains? How do the authors count organisms? Are two strains counted as two different organisms?

As described in the **Genome Sequence Datasets** of the **Methods** section, we selected one representative species per phylogenetic genus. In response to the reviewer’s comment, we have clarified throughout the manuscript that all references to the number of organisms refer specifically to the number of representative species included at the genus level in our dataset.

Line 289. The use of italics has gone wrong on this line.

The incorrect use of italics on this line has been corrected.

Line 481. Use lowercase 'c' for cellular.

The incorrect capitalization of the word “cellular” on this line has been corrected.

6. PLOS authors have the option to publish the peer review history of their article ([what does this mean?](https://track.editorialmanager.com/CL0/https:%2F%2Fjournals.plos.org%2Fplosone%2Fs%2Feditorial-and-peer-review-process%23loc-peer-review-history/1/010f0195804bf65a-d3176826-6941-48be-a0a7-a0ecdb8d5459-000000/l0xn7SBIZWlgaGikXSnj6-wFynOHP7JO-uLA59AWcME=201)). If published, this will include your full peer review and any attached files.

If you choose “no”, your identity will remain anonymous but your review may still be made public.

**Do you want your identity to be public for this peer review?** For information about this choice, including consent withdrawal, please see our [Privacy Policy](https://track.editorialmanager.com/CL0/https:%2F%2Fwww.plos.org%2Fprivacy-policy/1/010f0195804bf65a-d3176826-6941-48be-a0a7-a0ecdb8d5459-000000/lB-In1zgq4cFeA8L3SO549KXdH_zxxkra0Ip7x4-wkg=201).

Reviewer #1: No

Reviewer #2: **Yes:** David Studholme

[NOTE: If reviewer comments were submitted as an attachment file, they will be attached to this email and accessible via the submission site. Please log into your account, locate the manuscript record, and check for the action link "View Attachments". If this link does not appear, there are no attachment files.]

While revising your submission, please upload your figure files to the Preflight Analysis and Conversion Engine (PACE) digital diagnostic tool, [https://pacev2.apexcovantage.com/](https://track.editorialmanager.com/CL0/https:%2F%2Fpacev2.apexcovantage.com%2F/1/010f0195804bf65a-d3176826-6941-48be-a0a7-a0ecdb8d5459-000000/oAUUpLmzRjh14C1QuUD5zVdLSIvDWaxDj58nOVirUX8=201). PACE helps ensure that figures meet PLOS requirements. To use PACE, you must first register as a user. Registration is free. Then, login and navigate to the UPLOAD tab, where you will find detailed instructions on how to use the tool. If you encounter any issues or have any questions when using PACE, please email PLOS at [figures@plos.org](mailto:figures@plos.org). Please note that Supporting Information files do not need this step.
